# Managers' Perceptions of Factors Affecting Employees' Uptake of Workplace Health Promotion (WHP) Offers

**DOI:** 10.3389/fpubh.2020.00145

**Published:** 2020-05-05

**Authors:** Fanny Sigblad, Maria Savela, Leah Okenwa Emegwa

**Affiliations:** ^1^The Swedish Work Environment Authority, Stockholm, Sweden; ^2^Department of Public Health and Sport Science, Faculty of Health and Occupational Science, University of Gävle, Gävle, Sweden; ^3^Swedish Red Cross University College, Huddinge, Sweden

**Keywords:** workplace health promotion, managers, organization, employee, work-life balance

## Abstract

Managers are often charged with the responsibility of overseeing Workplace health promotion (WHP) for which significant amounts of resources are laid aside yearly. While there is increasing interest by employers to include WHP policies, studies show that WHP implementation and uptake by employees still need to be improved upon. Given that managers are part of organizational decision-making and implementation of new policies, they serve as the bridge between workers and management. The aim of this study is to investigate managers' perceptions of employees' WHP uptake as well as challenges encountered by managers in the execution of their WHP-related tasks.

**Method:** This study is based on a qualitative method using semi-structured interviews. Participants in the study were managers at medium and large-scale private companies in Northcentral Sweden. To ensure that participating companies are comparable in terms of structure and policy, only companies within the private sector were eligible to participate. Furthermore, only one manager per company was interviewed. A total of nineteen managers participated and the data generated were analyzed using content analysis.

**Results:** A total of three themes and nine subthemes emerged. The first theme deals with factors at the individual level, subthemes include awareness of WHP, work-life balance, and attitudes. The second theme comprises of factors related to the WHP offer, subthemes were design of the WHP, supportive collaborators and financing of WHP. The third theme deals with organizational factors, subthemes were the nature of the organization's operations, management as role models and resources and support for managers. Results show that most of the challenges encountered by managers in executing WHP were mostly at the organizational level.

**Conclusion:** Addressing modifiable factors at the individual and organizational levels and those related to the WHP may improve WHP uptake among employees.

## Background

The workplace is an important priority setting for population health promotion due to the significant amount of time spent at work (1, 2). Studies have shown the relationship between health, sickness absence, productivity and the economic growth of organizations including the importance of employee well-being for the individual, the organizations and society at large (2–4). These findings, coupled with various occupational health policies (5), have contributed to increasing awareness among employers regarding the implications of employee health (2). There are thus diverse policies and significant financial resources annually set aside for WHP (2, 6–8). Although most companies have policies and resources in place for WHP, they may be experiencing constraints in terms of implementation (6).

The Center for Disease Control and Prevention (CDC) describes WHP as coordinated and comprehensive efforts to enhance workers' health and safety (9). WHP strategies take the form of programs and policies for improving the physical work environment, minimizing risks at the workplace (9) as well as policies and benefits to create a “culture of health” (10, 11). Creating a culture of health often implies encouraging behavioral change and the adoption of healthy lifestyles such as smoking cessation, alcohol reduction, and increased physical activity, (10, 11). However, despite employers' positive disposition to WHP and the provision of wellness offers (12, 13), employees' uptake of WHP offers remains minimal (14, 15).

In Sweden, individual and group-based wellness offers aimed at promoting healthy lifestyles (e.g., increased physical activity) have been in workplaces since the 1970s (16). Depending on the worksite, common WHP strategies may include an in-house gym, monetary allowance (known as “friskvårdsbidrag”) for individual employees to engage in health promoting-activities outside of the workplace. Another popular WHP offer in Sweden is the “wellness hour” (i.e., employees may take 1 h off work per week to engage in health-promoting activities). Many of the offers are aimed at encouraging increased physical activity among workers. Physical activity is associated with general improvements in health behaviors and lifestyles, improved productivity, improved morale among employees, reduced absenteeism and economic gains for employers (17–21). A common practice among Swedish companies is the provision of monetary allowances to their employees (22). The monetary allowance is non-taxable and can be used for a range of specific activities e.g., membership at a gym (23). Swedish employment laws stipulate that WHP benefits should be provided to workers (23) however the amount and type of what is offered may vary across companies. Some stakeholders argue that non-uniform practices across sectors and professions may result in inequalities in access and utilization of WHP (24).

Managers are often charged with the responsibility of WHP in many organizations (25). They have a dual role as they are part of organizational decision-making but also responsible for the implementation of new policies (26) including WHP. Managers have closer contact with employees and serve as the link between workers and management. They play a vital role in policy implementation and in achieving desired changes (27). Unlike other forms of organizational policies, WHP involves an effort to encourage healthy lifestyle behaviors among employees (9). WHP is therefore sometimes perceived as an incursion into employees' private life and space, some stakeholders have expressed ethical and moral concerns about WHP (28). Given managers' role, known constraints to WHP implementation (6) and low levels of WHP participation among employees (14, 15), this study aims to examine managers' perceptions of factors affecting employees' WHP uptake. The study also intends to identify possible challenges that managers encounter in their WHP-related roles.

## Methods

### Study Design and Participants

The study is based on semi-structured interviews to understand managers' perceptions of employees' uptake of WHP offers and possible challenges related to WHP implementation encountered by managers. Companies were selected based on size and geographical location, i.e., only medium and large-scale companies operating within the Northcentral region of Sweden were eligible to participate. Small-scale companies, defined as those having <50 employees (29), were excluded. To ensure homogeneity in terms of policy and administration, only companies in the private sector were eligible to participate. Over 50 companies that met the criteria for size and geographical location were identified and initially contacted via email with a detailed description of the project and objectives. A follow up to the emails was later done through telephone calls, a total of nineteen companies indicated interest to participate.

Only one manager (per company) responsible for WHP and who has occupied that position for at least 6 months preceding the interviews were eligible to participate.

Data collection was conducted between November 2016 and January 2017 by an experienced research assistant with additional training specifically for this project. All interviews were conducted at a convenient location in each manager's workplace. The average length of an interview was about 33 min, the longest interview was 55 min. All interviews were recorded and later transcribed verbatim.

### Ethical Approval and Consent to Participate

Ethical approval for the study was granted by the regional ethical review board in Uppsala, Sweden. Before commencing each interview, participating managers were provided with information about the project and ethical aspects, including their right to withdraw participation at any time during the interview. Written consent was thereafter obtained, and interviews conducted.

### Data Analysis

The analysis method was inductive using content analysis. The transcribed interview material was studied and interpreted to identify patterns and themes and to have a deeper understanding of different phenomena (30). Content analysis is useful for investigating similarities and differences and for presenting results in a systematic and relevant manner (31).

The analysis was done in stages according to recommendations by Graneheim och Lundman (31). The first stage involved getting a holistic perspective of the data material, followed by the identification of meaning units related to the aim and objectives of the study. The text was then condensed to capture keywords and concepts that could systematically be marked as codes. To separate the content in the individual codes, they were compared for similarities and differences, similar codes were grouped and sorted into subthemes. Subthemes with similar contents were used to create main themes that reflected the meaning units (31).

## Results

A total of 20 managers, consisting of ten women and nine men aged between 36 and 66 years, were interviewed. They were mostly from construction, health care, food, and retail sectors. One of the interviews had to be excluded due to non-conformity to inclusion criteria. The results are therefore based on nineteen interviews, fourteen of these were from large scale companies and five from medium-sized companies. The participants had various job titles and roles such as Human resources (HR) manager, HR- specialist, HR- partner, personnel manager, and branch manager. Results from the interviews showed that participants generally believed that a good WHP plan contributes to a company's attractiveness. Various factors were however identified as affecting employees' uptake of WHP offers. Three themes and nine subthemes were identified. The main themes were individual-level factors, factors related to the WHP offer and factors at the organizational level. Managers encounter challenges in their WHP-related roles, but these were mostly at the organizational level. See Tables 1, 2 below for a summary of the data analysis process and themes, respectively.

**Table 1:** Example of the content analysis process from meaning units to themes, inspired by Graneheim & Lundman (31).

**Meaning units**	**Condensed meaning units (Description close to the text)**	**Condensed meaning units (Interpretation of the underlying meaning)**	**Code**	**Sub theme**	**Theme**
“…time constraint is a problem, people are of course at different stages of life. We have many employees who are parents of young children, these tend to not use this type of WHP offer because it is more difficult for them to find the time.”	Lack of time to utilize WHP due to having young children	Difficulty finding time for WHP after work due to having children to care for after work	Lack of time due to having younger children	Work-life balance	Individual factors
“…it is, of course, the amount of the monetary allowance that can be a hindrance…when you must add your own (money)…”	Out-of-pocket additional payments depending on amount offered	Likely low WHP uptake if employees must augment WHP offers with significant amount of money	Amount of Monetary allowance provided	Financing of WHP	Factors related to the WHP offer
“The most important factor is time. We are already experiencing quite a lot of time constraints and there is so much else to do, but at the same time we must think about (WHP)and employee well-being.”	Time constraints for WHP amid other responsibilities	Managers view WHP as additional responsibility to their primary roles. Adequate resources for executing WHP tasks (e.g., time) may therefore be lacking.	Managers lack time for WHP tasks	Resources and support for managers	Organizational factors

**Table 2:** Showing themes and sub-themes.

**Individual factors**	**WHP offers**	**Organizational factors**
- Awareness of WHP - Work-life balance - Attitudes to WHP	- Design of the WHP - Supportive collaborators - Financing of the WHP	- Nature of the organization's operations - Management as role models - Resources and support for managers

### Individual Factors

#### Awareness of WHP

Lack of awareness, often observed by managers through frequent requests for information, is common among new employees and younger staff. Below is how one manager described it:

”*…i believe that certain individuals are not aware of how much (monetary allowance) is available to staff and if wellness packages exist in the first place. We often notice this since we employ many people, many youths actually…”* (Respondent 20).

Regular and systematic information dissemination is important to counter uncertainty and lack of knowledge regarding the availability and administration of WHP offers. Some participants address the problem by “marketing” WHP offers during staff meetings, managers meeting, performance reviews, monthly newsletter, intranet and information brochures.

”* We have made it a recurrent point of discussion at all our departmental meetings. We talk about the importance of utilizing it…think we market it fairly well…”* (Respondent 18).

Some managers actively worked toward increased uptake of WHP through coaching, reminders, encouragement and personally inviting employees to try.

”*We can encourage them by visiting the worksites during staff meetings and talk about it…otherwise, I think that motivation and coaching in leadership are also about working more with health promotion and to get them to utilize WHP offers”* (Respondent 3).

#### Work Life-Balance

Most of the managers interviewed believe that individual employee's uptake of WHP offers depends on their life situation. Time constraints and tiredness were named as likely barriers:

”* …time constraint is a problem, people are of course at different stages of life. We have many employees who are parents of young children, these tend to not use this type of WHP offer because it is more difficult for them to find the time.”* (Respondent 5).

”* …for the wellness hour, it has been a case of me…skipping it if I have a lot to do. I do not use it because I have so much to do…”* (Respondent 15).

In companies where wellness hour is offered, employees have the option to close early from work, many managers allow flexibility to boost uptake.

#### Attitudes to WHP

Employees' individual attitudes and disposition to WHP may also result in low uptake of WHP offers. Specific examples named by managers include negative mindset, excuses, laziness, low commitment, declining interest, lack of motivation for necessary behavioral changes, non-prioritization of own health, and WHP.

”* …certain people are interested in this type of activity if I may say so. And some are less interested…it may, of course, be connected to the individual's lifestyle.”* (Respondent 20).

Employees who are convinced that they get enough physical activity in their daily commute to work and other motion may question the need for utilizing WHP offers. As one manager puts it:

”* …they (employees) think yes…, but do I still need to work out or use the exercise bicycle if I walk up 3 km a day? It's kind of not necessary, they say. They also think they get the needed physical activity from working”* (Respondent 17).

### Factors Related to the WHP Offer

#### Design of the WHP

Several elements related to the design and administration of the WHP offer were identified. A participatory approach, i.e., management's positive attitude to and support for staff initiatives, is believed to increase participation and foster improved relations between managers and staff. Management's sensitivity and responsiveness to individual employees' needs is important. They can, for example, conduct a survey to find out the types of wellness packages that are of interest to employees.

”* …One must, first and föremost, listen to the employee, what they want, what they think or feel. Decisions should be based on these. It is after all the employees that should be in focus since they are the ones who will eventually utilize the wellness offers…”* (Respondent 18).

It appears that WHP packages designed as group activities among coworkers often facilitate uptake because coworkers inspire and motivate one another. Examples of group activities are steps-count competition, team activities, recreational activities as well health-themed retreat away from the work environment.

”* …this type of step-count competition where all our 24 000 employees participate…it has shown effects just from last year till this year…just this wellbeing and people losing weight, feeling better and so on…”* (Respondent 17).

”* So, a combination of one being able to use their wellness allowance individually …and additional group activities just like the company does, makes more people start. And it has, from an organizational point of view, felt like an incredible success on our part…it has been a combination of (WHP offers at) the individual level… and group level, it has increased uptake” …* (Respondent 18).

Some managers stressed the need for inclusiveness i.e., WHP should be available to all workers irrespective of their employment status

”* …we do not differentiate, every worker gets their WHP monetary allowance, irrespective of whether I am part-time or permanent staff, I get the same WHP allowance”* (Respondent 14).

#### Supportive Collaborators

Enlisting the help of external and internal supportive collaborators were judged as positive for improving uptake of WHP offers. Examples of external collaborators are wellness and fitness centers, health coaches, personal trainers, occupational health consulting firms. These partnerships become relevant for wellness-themed organizational meetings and activities, on-site fitness training and lectures on health and wellness.

”* …(we) have a gym chain that is connected to wellness allowance…we have had that in a project. Personal trainers and fitness experts from the gym chain were at our worksite to inspire and speak about what one can do…”* (Respondent 10).

”*… we had a wellness intervention in which we got an occupational health consulting firm to come to our worksite and conduct this kind of tests, fitness test as it is called…the result is that we have 2–3 additional employees who now train and engage in physical activities more regularly”* (Respondent 9).

For best results, external collaborators should not be located too far away and should offer a wide range of alternatives for employees to choose from. Unfortunately, employees in smaller cities are often unable to access WHP offered by collaborators whose facilities are concentrated in large cities. Moreover, the routine for companies who offer a fixed monetary allowance is that employees make out-of-pocket payment, but get a refund up to the amount offered by the company. To avoid the extra financial burden of out-of-pocket payments, managers suggest delivering WHP offers through systems and portals that are easy to access, especially those connected to wellness centers.

Internal collaborators were in the form of “Health Peer Educators.” These are employees in the same organization who volunteer to disseminate information about WHP offers. They plan activities aimed at getting colleagues interested in WHP and keeping them motivated to utilize WHP offers.

”* …we have about 30–35 health peer educators who are also part of our health council. Their role is to spread information regarding what activities we have, spread information about wellness allowances…initiate and participate in activities…”* (Respondent 16).

Some managers were critical of the lack of systematic evaluation and follow-up of WHP interventions:

”* …We have no follow-up whatsoever, we simply get a receipt that (the employee) has paid. But the question is: does he really go for these activities or are we simply giving money to the gym? Has it added any value to us as a company? So, it is a bit questionable that we do not have any follow up whatsoever”* (Respondent 16).

#### Financing of WHP

According to managers, low uptake was common for WHP offers for which employees must make an additional out-of-pocket payment. Managers reported getting regular requests from staff for an increase in the fixed-rate monetary allowance offered, uptake seem to improve when the amount was increased.

”* …it is, of course, the amount of the monetary allowance that can be a hindrance…when you must add your own (money)…”* (Respondent 15).

An increase in the subvention offered to employees led to increased WHP uptake at one of the participating worksites:

”* …the amount was increased by 300% so that we have a fairly large amount of money, one can then appreciate the benefit of utilizing the allowance. It covers yearly membership at a fitness center, and it covers quite a lot, therefore there has been an increase in employees' interest to utilize it, I think”* (Respondent 14).

”* …we created better economic conditions for employees to be able to purchase membership at fitness centers or swimming centers or whatever else. This is because I think financial constraint is often the problem …”* (Respondent 15).

On the contrary, one manager believed that it is a positive approach to offer only subsidized WHP offer, that way employees can complete with own funds:

”* …I always believe in people having to pay a little (for WHP). It means they will be more committed to the services purchased (e.g., gym membership)”* (Respondent 18).

### Organizational Factors

#### Nature of the Organization's Operations

Factors such as irregular working hours, strenuous tasks, job description and non-uniform organizational structures across branches may limit employees' uptake of WHP offers:

”*Actually, it is the nature of the company's operations that demand that I have such and such role or such position… makes it difficult for me to choose the wellness hour… it is clear that your role or position can be a limitation”* (Respondent 6).

”* …one of the biggest barriers is that we are mobile, we never work within four walls, it keeps changing…”* (Respondent 9).

Suggestions to address barriers include the provision of on-site gym and duty rosters that accommodate participation in WHP even during working hours:

”* …this might sound stupid, but I think the only way out is to incorporate wellness into working hours…i doubt we can make this work if it is not within working hours”* (Respondent 16).

”* concerning that, if every company should own a gym or a treadmill or exercise bikes at the work site…it would be easier to engage in physical activity. Or having a fitness center very close by to the company”* (Respondent 18).

#### Management as Role Models

Managers believed that top management's uptake of WHP offers would set a good example and encourage employees to utilize theirs. By utilizing WHP offers, top management staff would reinforce the importance of health promotion and show their readiness to influence the entire organization in the right direction.

”*…a lot has to do with being a role model, to be an ambassador for what is available… because if I, as boss, utilize and speak well of available wellness offers then the information will spread through the entire organization and the wellness offers will be viewed as good…”* (Respondent 15).

”* …so, I think that top management has to view it (WHP) as important and there must be follow up too…more resources will be put in place if top management considers it as important”* (Respondent 16).

#### Resources and Support for Managers

Time constraint, the responsibility to convince top management and the constant need to provide proof that investing in WHP can result in measurable economic gains for the company are major barriers encountered by managers.

”* The most important factor is time. We are already experiencing quite a lot of time constraints and there is so much else to do, but at the same time we must think about (WHP)and employee well-being”* (Respondent 7).

”* In fact, one must calculate and show in figures the whole time, it is always the economy first. We must always show them the costs and long-term benefits in clear detail…they must understand that it is better to invest in health promotion rather than rehabilitation. We must show the top management these things in figures”* (Respondent 3).

Respondents raised the need for companies to have a managerial position that deals mainly with WHP. A manager with such a role must possess a holistic view and understanding of WHP, must have adequate time for WHP and support function for management in their decision-making.

”*…I think it has a lot to do with the fact that one has never before had a human resource function with a holistic view of the organization …It is important to have an overall health coordinator who deals with health and health promotion issues…”* (Respondent 3).

Some respondents spoke about the need for sending managers on training courses so that they may improve their knowledge of occupational health promotion. Training will ensure that managers have enough skills for motivating and supporting all categories of employees, including those with diverse needs.

”* The most important thing is training for managers so that they are well-equipped to get out the best in their workers…and how to improve as leaders…”* (Respondent 3).

## Discussion

This study investigated managers' perceptions of factors affecting employees' uptake of WHP offers. Three themes and nine sub-themes were identified, i.e., factors related to the individual (with sub-themes: awareness of WHP, work-life balance and attitudes); those related to the WHP (sub-themes: design and implementation of WHP, supportive collaborators, and financing of WHP) and factors at the organizational level (sub-themes: nature of the organization's operations, management as role models and resources, and support for managers).

Although an individual's autonomy in decision making is an important ethical principle in health promotion (32), identifying individual-level barriers and addressing them are important for improving WHP uptake. Lack of awareness and negative attitudes are modifiable factors that may improve uptake without infringing on employee's autonomy. Issues of work-life balance, such as the time constraints experienced by employees with diverse life situations, are crucial for uptake. Work-life balance is used to describe the impact of work and family life on working individuals. The concept has its history in policy efforts to reduce the effects of gender inequalities and low female labor participation due to caring for young children (33). The concept is now used more commonly in recognition of the various meaning of family challenges beyond caring for children. According to Kossek et al. (34), employers can work toward a sustainable workforce by taking into cognizance the relationship between employees' well-being and work-life balance. To address individual-level factors, managers in this study adopted various strategies such as flexible “wellness hour,” targeted information dissemination, site visitations, individual coaching and creating opportunities for employees to ask questions during staff meetings. These are laudable strategies as research has shown that employees' engagement in WHP improves when they perceive strong organizational support and involvement (35).

Factors related to the WHP offer were WHP design, supportive collaborators and financing. A participatory approach, that encourages and supports employees' WHP initiatives, was viewed as a key factor for improving WHP uptake. A possible explanation is that employees' initiatives may reflect their actual needs rather than what management perceives as important. According to the Centers for Disease Control and Prevention (36), effective WHP is built on a continuous process of needs analysis, priority setting, planning, implementation, and evaluation. Our finding is in line with those of Kilpatrick, Blizzard, Sanderson et al. (37), who found that participation in WHP was higher among workers who felt consulted and those who perceived other colleagues as engaged and interested in WHP.

The success of WHP was perceived to be partly related to external and internal collaborators and optimal administrative systems for WHP. Supportive external collaborators are, for example, employee assistance program providers (EAP), fitness chains, wellness companies, among others. Many of these external collaborators have portals that offer employees easy access and a variety of activities to choose from. However, although engaging external collaborators was generally viewed as an enabling factor, managers believed that the success rate is dependent on the type of collaborator, the range of activities provided and the location of the WHP. Many of the managers reported that their company's current external collaborators mostly had facilities in larger cities. This is problematic for employees residing in or working in branches located in smaller towns, they may find it difficult to access or participate in activities located far away. A recommendation is that large corporations with branches in both large and small cities, but who operate a centralized WHP policy may have to decentralize certain aspects of their WHP policies and structure. Without proper evaluation, it is difficult to judge the efficiency of external collaborators. Managers were critical about the continued engagement of external collaborators without regularly evaluating outcome or impact. In a study by Compton & McManus (38), it was found that the non-evaluation of external collaborators is a general problem and must be addressed.

The effectiveness of strategies to create a culture of health and good communication at workplaces (39) was further confirmed in the present study. Apart from managers strategies to spread information, some companies have employees who volunteer to be on the organization's health council as “health motivators.” They help to raise awareness about health and WHP by spreading information to their colleagues. Although information may be available on company websites and WHP portals, employees are less likely to seek out such specific information. The creation of a health motivator role is, therefore, an innovative way to ensure adequate information dissemination and keeping employees motivated to achieve the organization's WHP goals.

Findings from the interviews showed that how WHP is financed is important for uptake. According to Swedish employment laws, WHP benefits should be provided to workers (23) but the amount and type of what is offered vary across companies. A common practice among employers is the provision of monetary allowance for individual employees to engage in a health promotion activity of their choice. The tax agency provides a list of a wide range of activities on its website. Managers had opposing views about the role of partly financed WHP offers requiring out-of-pocket payment by employees. While some managers viewed this as a barrier (i.e., financial constraints will result in low uptake), others considered it as an enabling factor (i.e., out-of-pocket payment by employees will lead to increased uptake and commitment to WHP). It is unclear whether these contrasting views are as a result of individual differences between managers or due to contextual differences in the workplaces they represent. Contextual differences such as the size of the monetary allowance and therefore, the amount of out-of-pocket-payment to be made, may be responsible for the differences in opinion among managers.

Unfortunately, the above variation may create possible inequalities in WHP offers and consequently uptake among employees in different organizations, sectors, and professions. Such inequalities may be larger when comparing larger organizations with smaller organizations (6). Although the size and financial capacity of individual companies must be taken into consideration, the importance of a systematic and continuous process of needs assessment, priority setting, planning, implementation, and evaluation cannot be ignored (36). The assumption that out-of-pocket payment to complement monetary allowance is positive for uptake may reflect a general approach to WHP. Employers probably design WHP based on an assumption that they know employees' needs and without consulting the employees. A participatory approach when designing WHP is useful for focusing on areas identified by employees, which is likely to improve uptake.

Although individual factors and factors related to the WHP were identified, it appears that organizational factors were crucial to what happens at the individual and WHP levels. For example, the finding that many managers still struggle to convince top management about investing in WHP probably suggests a lack of understanding of the importance of WHP among top management in some organizations. It also appears that despite growing awareness and interest for WHP among employers, they are probably hesitant to invest in WHP beyond a certain basic level. It is also likely that some employers do not view WHP to include life outside of the workplace or as encompassing psychosocial health and well-being. Likely reasons could be due to a narrow perception of WHP to only mean on-site strategies like accident reduction, a notion that has been observed especially in rural settings (40). Similarly, some managers lack adequate organizational support and resources for WHP (e.g., time and training). Challenges faced by managers may be better addressed if management is interested in developing a well-structured and goal-driven WHP policy. Many of the managers have come to understand the importance and intricacies of WHP to the extent that they suggest the creation of managerial positions with a specific focus on WHP.

Managers need adequate training and skills for the successful execution of WHP related tasks. Other organizational level challenges faced by employees include irregular working hours, strenuous and limiting job tasks, jobs that involved high mobility, non-uniform organizational structure in many large corporations. Strategies suggested to address such barriers include permitting wellness activities during working hours and flexible work scheduling among others. The above suggestions from managers can only be considered if top management has the right perspective concerning WHP i.e., what it is and how it works. The concept of health-promoting leadership is thus an emerging term that describes leadership behaviors for health-oriented organizations (41). According to Erikson (41), health-promoting leadership includes the systematic health-oriented development of the physical and psychosocial work environment. Some previous studies have shown that the success of WHP intervention depends, not only on the structure of the intervention provided but also on organizational involvement (19, 42).

Although WHP offers are common in Sweden, this study is one of the few that have explored factors related to employees' uptake of WHP from managers' perspectives, as well as challenges encountered by managers responsible for WHP. Some methodological issues are worth highlighting. The study is based on interviews of a convenient sample of managers from organizations willing to participate in the study, results are therefore not generalizable. As only private organizations were included in the study, it is likely that WHP in the public sector works differently compared to the private sector. Moreover, considering that managers had differing opinions regarding certain issues, an analysis of factors likely to explain this difference would have been appropriate. For example, it would be relevant to see if the differences in opinion were associated with gender, age, years of experience, the size of their company, the type of WHP offered, just to name a few. Overall, this study provides an insight into WHP uptake among employees in private medium and large-scale companies, challenges faced by managers and modifiable factors to address challenges and improve uptake.

## Conclusions

Findings from this study show that employees' uptake of WHP offers can be improved by addressing certain factors. To address factors at the individual level, organizations should regularly inform employees about WHP offers. This will increase awareness, generate interest and keep employees motivated. The wellness-hour and increased flexibility around it may be a good strategy to address constraints related to work-life balance. Further research is however needed to investigate its effectiveness and impact. The design and delivery of WHP can be improved by adopting a participatory approach and regular evaluation of WHP practices. A regular evaluation will ensure that organizations are able to measure outcomes, identify areas of unmet need and improve where necessary. To prevent exclusion and inequality in access to WHP, there should be alternatives for small-town dwellers who may be unable to access city-based WHP facilities. Another example is to determine the implications of WHP financing and employees' out-of-pocket payments and how they affect WHP uptake. At the organizational level, the role of providing adequate training and support in terms of resources and time for managers can not be overstated. WHP should not be treated as a side responsibility in addition to managers' primary role. For effective implementation, enough time and resources should be allocated to managers. Management should show interest by also utilizing WHP and ensuring that WHP designs match the nature of the company's operations. For example, “a one-size fits all” kind of approach will be ineffective in organizations with many workers whose job tasks involve high mobility outside of the office.

In conclusion, WHP is a common feature of many Swedish workplaces. Given the amount of resources annually spent on WHP, the need for establishing evidence (or lack of it) for WHP's effectiveness and impact can not be overemphasized. Further research that uses other methods and includes diverse participant categories e.g., the public sector is warranetd if results are to be generalized to the rest of the population.

## Data Availability Statement

The datasets generated for this study are available on request to the corresponding author.

## Ethics Statement

The studies involving human participants were reviewed and approved by the regional ethical review board in Uppsala, Sweden. The patients/participants provided their written informed consent to participate in this study.

## Author Contributions

LO and MS conceived of the project idea, designed the study, and coordinated data collection. FS and LO were involved in data analysis and manuscript drafting. LO finalized the manuscript.

## Conflict of Interest

The authors declare that the research was conducted in the absence of any commercial or financial relationships that could be construed as a potential conflict of interest.
